# Effects of contoured insoles with different materials on plantar pressure offloading in diabetic elderly during gait

**DOI:** 10.1038/s41598-022-19814-0

**Published:** 2022-09-13

**Authors:** Qiu Qiong Shi, Pui Ling Li, Kit-Lun Yick, Nga-Wun Li, Jiao Jiao

**Affiliations:** 1 School of Fashion and Textiles, The Hong Kong Polytechnic University, Hong Kong, China; 2 Laboratory for Artificial Intelligence in Design, Hong Kong, China; 3 Dr. Stephen Hui Research Centre for Physical Recreation and Wellness, Hong Kong Baptist University, Hong Kong, China

**Keywords:** Health care, Materials science

## Abstract

To investigate the effect of contoured insoles constructed of different insole materials, including Nora Lunalastik EVA, Nora Lunalight A fresh, Pe-Lite, and PORON Medical 4708 with Langer Biomechanics longitudinal PPT arch pads on offloading plantar pressure on the foot of the elderly with Type 1 or 2 diabetes during gait. Twenty-two elderly with Type 1 or 2 diabetes participated in the study. Their plantar pressure was measured by using an insole measurement system, while the participants walked 10 m in their bare feet or used each experimental insole in random order. The plantar surface was divided into four specific regions including the toes, forefoot, midfoot, and rearfoot. The mean peak pressure (MPP) and pressure–time integral (PTI) of ten steps with or without wearing one of the four insoles were analyzed on the dominant foot and the four specific plantar regions. After completion of the activities, the participants scored each insole from 1 (the least comfortable) to 10 (the most comfortable). The analysis of variance (ANOVA) factor of the insoles had significant effects on the MPP (*P* < 0.001) and PTI (*P* = 0.004) in the dominant foot during gait. Pairwise comparison results showed that the MPP and PTI in the dominant foot were significantly lower (*P* < 0.001) with PORON Medical 4708 than barefoot, Nora Lunalight A fresh, and Pe-Lite. Additionally, the insole materials had a significant effect for the forefoot (*P* < 0.001) and rearfoot (*P* < 0.001) in terms of the MPP and PTI compared with the barefoot condition during gait. Regardless of the plantar region, the MPP and PTI values were the lowest when PORON Medical 4708 was used as the insole material among four insole materials. Meanwhile, a significantly lower MPP and PTI can be found in the forefoot and rearfoot with the use of the four experimental insoles when compared with barefoot. The soft insole materials (i.e., PORON medical 4708 and Nora Lunalastik EVA) had a better performance than the rigid insole materials (i.e., Nora Lunalight A fresh, and Pe-Lite) on plantar pressure offloading for diabetic elderly.

## Introduction

There were an estimated 537 million adults (20–79 years old) globally with diabetes in 2021. The International Diabetes Federation predicted that this number will increase to 643 million by 2030 and 783 million by 2045 International Diabetes^1^. The majority (75%) of diabetic patients are 45 years old and older^2^. Diabetic foot ulcers (DFUs) are one of most serious complications for diabetic patients^3^. DFU issues affect one in three people with diabetes^4^, and the rapid increase in the prevalence of DFUs over the last few decades is a major challenge for healthcare systems around the world^5^.

The peak pressure value is defined as the highest pressure and frequently reported in plantar pressure studies^6–9^. Increases in peak pressure have been found to be particularly related to the development of DFUs due to the presence of peripheral neuropathy^10,11^. Both Types 1 and 2 diabetic patients are at risk of developing diabetic peripheral neuropathy due to uncontrolled or long-standing chronic hyperglycemia^12^. Many of the elderly with diabetes show an increase in peak pressure during gait^13^. The high levels of peak pressure show that the pressure has a linear regression relationship with DFUs in diabetic patients especially with peripheral neuropathy^14^. Due to higher magnitude and duration of mechanical stress within the foot during standing and walking, abnormal plantar pressure distribution on the soft tissues of the feet can result in soft-tissue breakdown and subsequent foot ulcers, limited joint mobility, and foot deformities in diabetic foot pathology^12,15–17^. Such deteriorations increase the risk of falls and disability^17^, and cause a higher rate of mortality and worse life quality of the elderly with diabetes^18,19^. Therefore, evaluations of the peak pressure of the feet of diabetic patients have been commonly done to determine the effectiveness of footwear and foot devices (i.e., medical insoles, shoes, and orthoses) in relation to pressure offloading^12,20^. The effectiveness of diabetic footwear is determined by a significant reduction or lower peak pressure value^21^. Pressure–time integrals (PTIs) are also commonly reported and used in DFU studies^3,12^. The PTI is defined as the area under the peak pressure time curve^7^. The PTI is considered as an essential parameter of DFUs because it incorporates pressure and time factors^21^. Duan et al.^3^ showed that diabetic patients have a normal plantar blood flow response after walking at a low PTI stress, but impaired blood flow response after walking at a high PTI stress. This finding indicates that it is important for diabetic footwear products to prevent diabetic patients from walking with a stimulus with a high PTI.

Some clinical studies suggest that usage of suitable orthotic footwear or insoles that offer proper arch support can effectively offload plantar pressure^22–25^, which is an essential intervention to prevent the risk of DFUs^26–29^. Soft materials can readily be moulded for a custom fit to offer excellent wear comfort and cushioning of the feet^30^, while a composite structure constructed of soft and rigid materials have a superior performance in reducing the amount of plantar pressure^31,32^. In addition, the use of an arch support with an insole has been proven to offload plantar pressure when compared to an insole without an arch support for patients with diabetes^33–37^. The type of foam material used is also subjectively determined based on the experience of individual practitioners. Cellular polymer such as polyurethane foam (e.g., PORON) is an open-celled material with excellent cushioning properties^16,30,31^, while ethylene–vinyl acetate (e.g., nora) is moldable, resilient and elastic^30^ so that insoles that use this material can prevent callus formation and tissue damage because the material exerts less frictional force and shearing stress to the skin^16,30,35–38^; Polyethylene (e.g., Pe-Lite) offers good cushioning and shock absorption^39^. Lo et al.^38^ tested the physical properties of those insole materials (e.g., polyurethane, ethylene–vinyl acetate, and Polyethylene), but lacked practical clinical evidence for plantar pressure offloading. All of these materials are also available in a wide range of hardness, thickness and densities. For optimal foot protection, custom-fabricated orthotic insoles are typically made by using multiple layers of materials so as to provide the desirable amount of support and reduction of peak plantar pressure and PTI for diabetic patients^16,31^.

Despite a large volume of biomechanical studies that have documented how insoles alleviate plantar pressures, there are large variations in the type of material used, shape, construction and properties of the insole and footwear conditions. Due to the lack of evidence in the literature, current understanding on the approach to selecting the type of insole material used to optimize the offloading performance is somewhat limited. The fabrication of insoles still greatly depends on repeated trials and error based on the experience of practitioners, and the availability of materials locally, rather than the foot conditions of patients and effectiveness of offloading. Hence, the aim of this study was to investigate the effectiveness of the above-mentioned soft insole materials (i.e., PORON medical 4708 and Nora Lunalastik EVA) and the rigid insole materials (i.e., Nora Lunalight A fresh, and Pe-Lite) on offloading the plantar pressure of the elderly with diabetes during gait. The MPP and PTI of the dominant foot and four different plantar regions (i.e., toes, forefoot, midfoot, and rearfoot) were measured, and comparisons of MPP and PTI with or without the use of the different insoles were made. The influence of the type of insole material used on plantar pressure distribution was systematically investigated. Effectiveness in plantar pressure offloading by insole materials was determined in terms of significantly lower values of MPP or PTI, and vice versa. It was hypothesized that the contoured insoles constructed of different insole materials used in this study would offload plantar pressure than barefoot for diabetic elderly during gait, especially for the forefoot and rearfoot regions.

## Materials and methods

### Participants

Human subject ethics approval was granted by The Hong Kong Polytechnic University Ethics Committee (HSEARS20200128001) for this study. All methods were performed in accordance with the relevant guidelines and regulations. Twenty-two (13 men and 9 women) diabetic patients (age: (mean ± SD) 62.6 ± 5.5 years old; height: 164.3 ± 6.0 cm; body mass: 65.0 ± 11.3 kg; body mass index: 24.1 ± 3.4 kg/m^2^; duration of diabetes: 8.9 ± 8.3 years; diabetes type: Type 1 (8 participants), Type 2 (14 participants); hemoglobin A1c: 7.0 ± 0.9%); shoes size: EU39.2 ± 1.9 (female), EU41.3 ± 1.0 (male)) were recruited to take part in the study. The sample size was calculated by using G * power^16^, for a two-tailed test with 1 − β = 0.08, and α = 0.05 with five groups. The estimated total sample size was twenty participants. To be more conservative, twenty-five participants were recruited in this study for avoiding any drop out and ensuring the estimated sample size can be obtained. Three participants dropped out during study due to their time schedule problems. The informed consent forms were obtained from all participants prior to the experiment after a briefing on the procedures. The inclusion criteria were: (a) age between 50 and 70 years old, and (b) a diagnosis of diabetes (Type 1 or 2). The exclusion criteria were: (a) balance impairment, participants’ static and dynamic balance abilities were assessed by Berg Balance Scale^40^ under the therapist; (b) assessed to be at high risk of falls; (c) impaired gait when participants were unable to walk along a 10-m walkway at least five times without assistance^41–43^; (d) blood pressure higher than 130/90 mmHg^44^; and (e) foot ulcer or history of food ulcer.

### Experimental insoles

**Figure 1:**
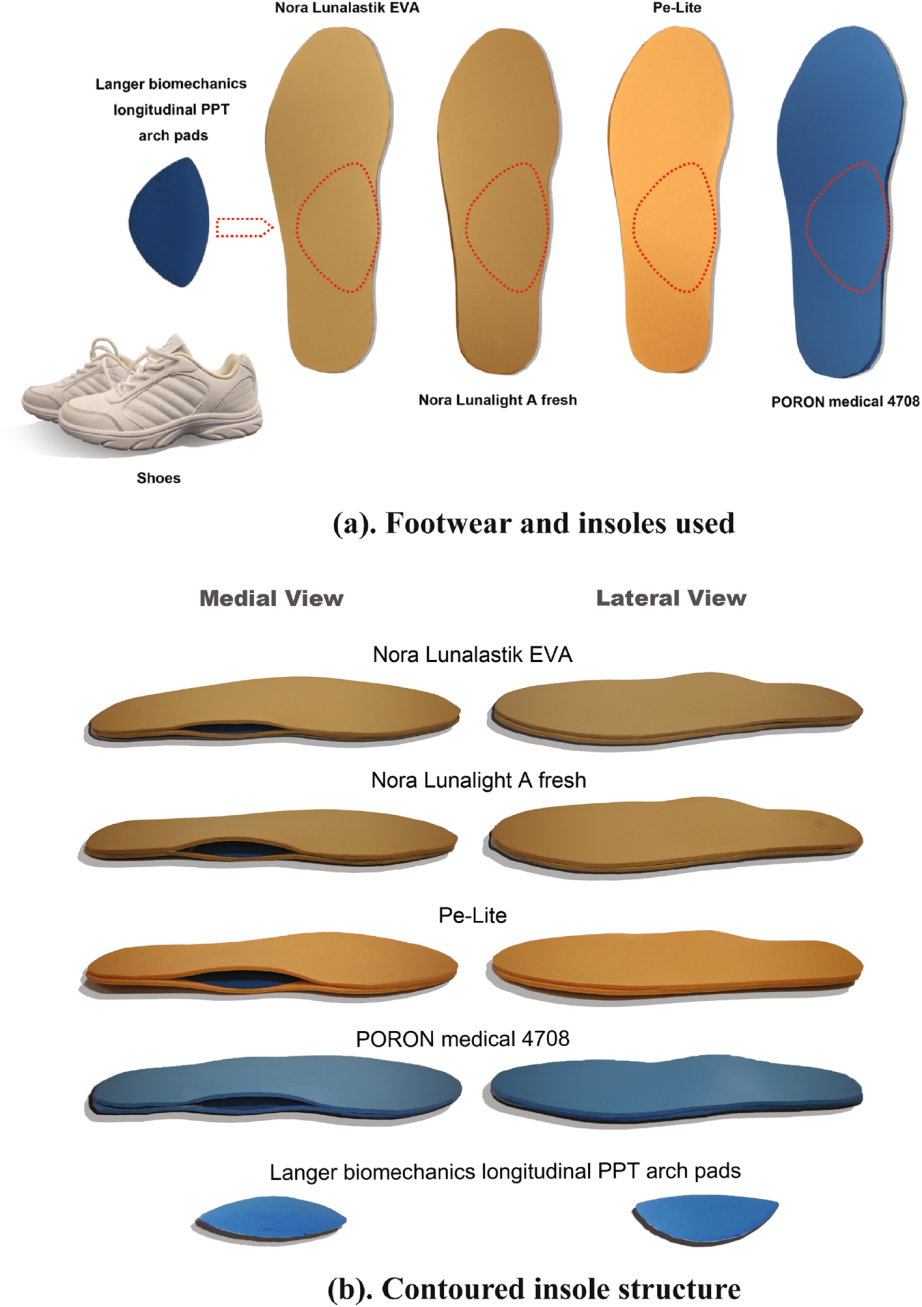
(**a**) Footwear and insoles used. (**b**) Contoured insole structure.

The materials used in this study are presented in Fig. 1a. The experimental insoles were cut-out from insole materials, including rigid materials and soft materials (Nora Lunalastik EVA, and PORON medical 4708). The same type of leather shoes (Kinghealth, Hong Kong) was given to each participant. During the experiment, each insole used has a contoured two-layer structure (see Fig. 1b) with the Langer Biomechanics longitudinal PPT arch pads inserted between each layer. The original thickness of each layer of the insole material, the Langer Biomechanics longitudinal PPT arch pads, and contoured insole were measured by using a digital tester (KFG-2021). Then the simulated thickness of each two-layer insole, and the thickest part of the experimental contoured insole during weight-bearing walking were measured under compression stresses of 50 kPa, 150 kPa, and 200 kPa by using an INSTRON 4411 tensile strength tester^45^. Table 1 shows that the original thickness of the materials, and the changes in thickness of each insole material due to their different hardness.

**Table 1:** Basic physical properties of studied insoles.

Sample (2-layer insole)	Thickness (initial) (mm)	Thickness under 50 kPa (mm)	Thickness under 150 kPa (mm)	Thickness under 200 kPa (mm)	Density (g/cm^3^)	Hardness
A	8.20	8.20	8.18	8.16	0.35	Rigid 58 shore A
B	8.00	7.95	7.88	7.79	0.16	Rigid 30 shore A
C	6.20	6.01	5.48	5.04	0.23	Soft 25 shore A
D	6.05	5.71	4.58	3.51	0.20	Soft 18 shore A
E	8.20	7.05	5.53	4.48		

A—Nora Lunalight A fresh; B—Pe-Lite; C—Nora Lunalastik EVA; D—PORON medical 4708; and E—Langer Biomechanics longitudinal PPT arch pads.

### Plantar pressure measurement

The plantar pressure values were obtained by using a reliable and accurate insole measurement system—Pedar with 99 sensors (Novel GmbH, Munich, Germany)^46,47^ to evaluate the four insoles during walking. The capturing frequency is 50 Hz per second. Calibration was conducted according to the manufacturer instructions before each trial. Pedar sensors of the appropriate size were inserted into the shoes and placed on the top of the insoles, after which the participants underwent a biomechanical plantar pressure analysis in five conditions (i.e., barefoot, and using the four different insoles) on a straight 10 m walkway with self-selected walking speed to perform their normal gait characteristic^8^, their average walking speed was 6.1 ± 1.2 m/s. For barefoot condition, the proper size Pedar sensors were attached with plantar and fixed by sock wearing without shoes. Each experimental insole was worn in a randomized order (see Supplementary Table S1). The participants repeated the series of gait trials until 10 acceptable consecutive steps were obtained.

**Figure 2:**
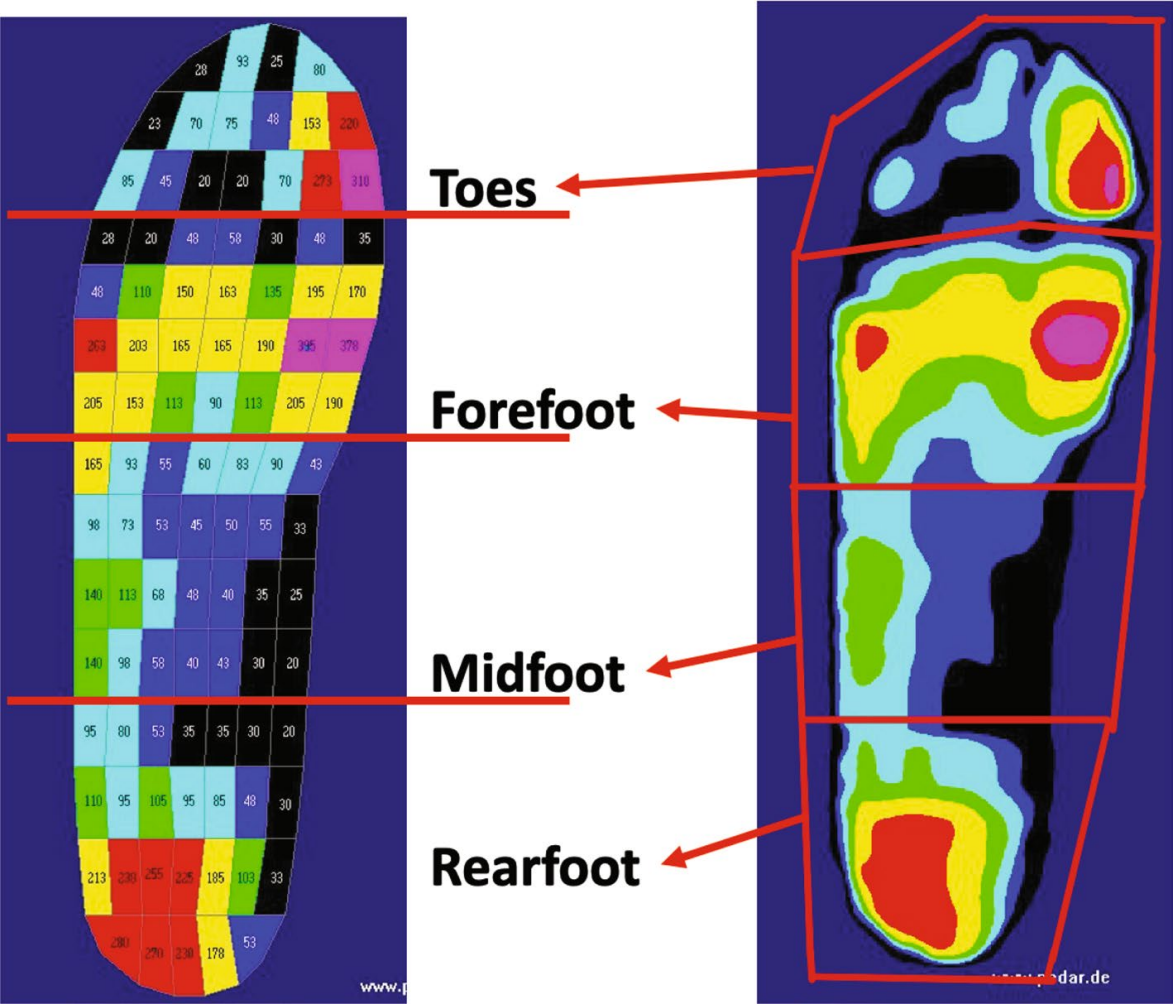
Four specific plantar regions.

The plantar surface was divided into four specific regions including the toes, forefoot, midfoot, and rearfoot (Fig. 2)^16,48,49^. The MPP and PTI of the ten steps with or without using the experimental insole were obtained from the Pedar system, and then the data were analyzed based on the dominant foot, and the four specific plantar regions. The barefoot results were also compared and analyzed as reference. The dominant foot was determined by the procedure in which foot was used to kick a ball^50^. After conducting the experiment, the participants scored each insole from 1 (the least comfortable) to 10 (the most comfortable).

### Statistical analysis

**Figure 3:**
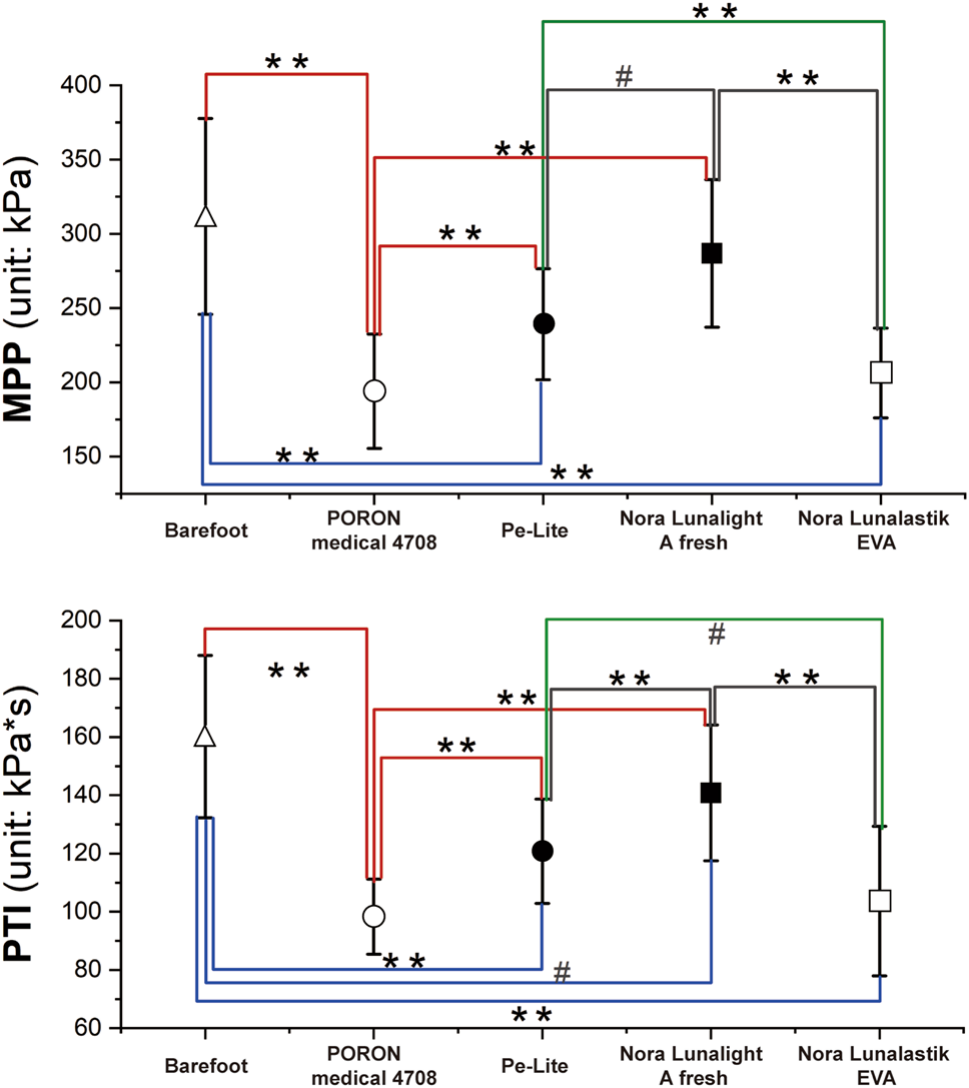
MPP and PTI values of dominant foot. *Notes:* ^**#**^*P* < 0.05 and **P < 0.001.

**Figure 4:**
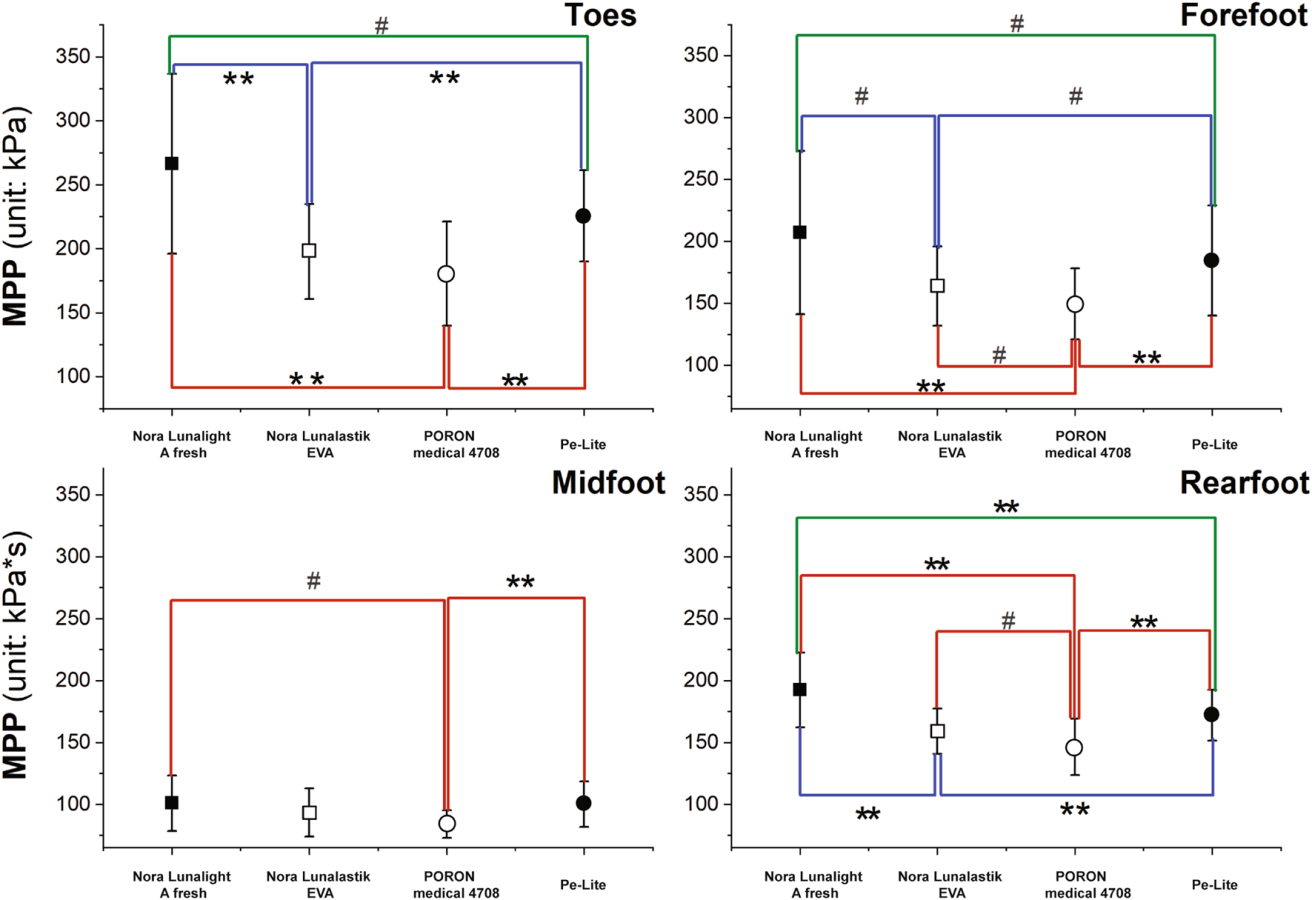
MPP in four different plantar regions with use of four insoles. *Notes:* ^**#**^*P* < 0.05 and **P < 0.001.

**Figure 5:**
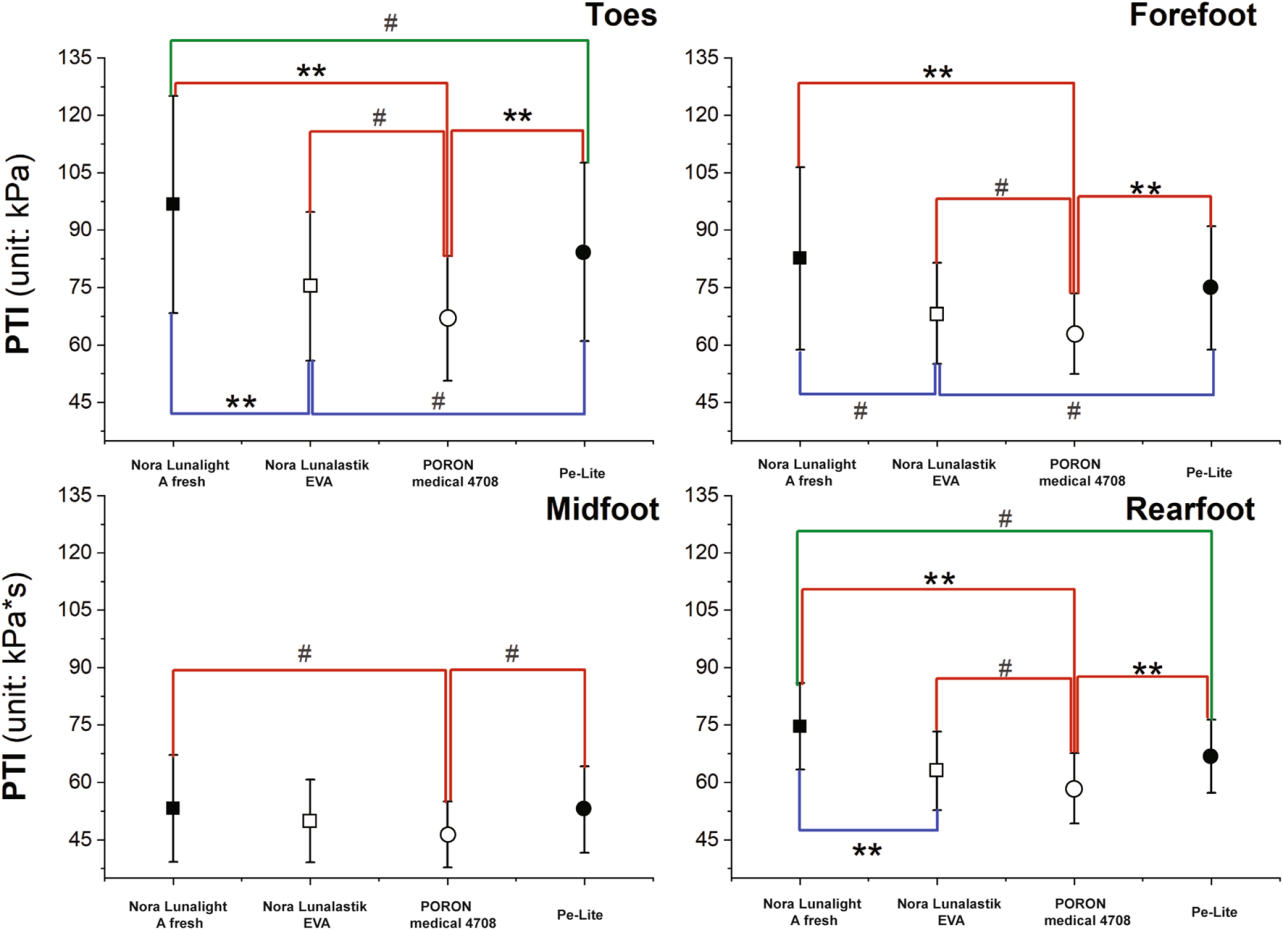
PTI in four different plantar regions with use of four insoles. *Notes:* ^**#**^*P* < 0.05 and **P < 0.001.

SPSS software (SPSS Statistics IBM, Version 20.0) was used for the statistical analysis. The Kolmogorov–Smirnov test was used to determine the normality of the distribution of the data. Then a student *t* test was applied to see if significant differences exist between the dominant and subdominant foot on MPP and PTI. If no significant difference was found between the foot, a within-subject analysis of variance (ANOVA) with one-way repeated measures was employed to examine the insole effects of the dominant foot. Statistical significance was set at *P* < 0.05, and a significant level of *P* < 0.001 is also indicated in Table 2 and Figs. 3, 4 and 5. If significance was found, a pairwise comparison of the differences between conditions for those variables was employed through post hoc testing with Bonferrroni. The effect size was quantified by using partial eta squared (${\upeta }_{p}^{2}$). All the data are presented as mean and standard deviation (SD) (i.e. mean (SD)) in the tables and figures.

**Table 2:** Statistical results of MPP and PTI with and without insoles (mean (SD)).

Plantar region	Barefoot	PORON Medical 4708	Pe-Lite	Nora Lunalight A fresh	Nora Lunalastik EVA	*P* value	${\upeta }_{p}^{2}$
**MPP (kPa)**							
Toes	169.6 (64.6)	180.6 (40.7)	225.7 (35.6)^**#**^	266.3 (70.4)**	197.8 (37.1)	< 0.001**	0.869
Forefoot	306.2 (68.3)	149.6 (28.6)**	184.5 (44.3)**	207.1 (65.8)**	164.1 (32.0)**	< 0.001**	0.934
Midfoot	106.5 (40.2)	84.0 (11.1)	100.1 (18.2)	100.9 (22.3)	93.3 (19.4)	**0.002**^**#**^	0.603
Rearfoot	266.6 (43.4)	146.4 (22.7)**	171.9 (20.6)**	192.4 (30.1)**	159.0 (18.2)**	< 0.001**	0.923
PTI (kPa*s)							
Toes	54.5 (22.4)	66.9 (16.3)^**#**^	84.3 (23.3) **	96.7 (28.4)**	75.3 (19.4)**	< 0.001**	0.786
Forefoot	114.0 (29.8)	62.9 (10.5)**	74.9 (16.1)**	82.6 (23.8)**	68.2 (13.2)**	< 0.001**	0.845
Midfoot	46.8 (18.0)	46.4 (8.6)	52.9 (11.3)	53.2 (14.0)	49.9 (10.8)	**0.012**^**#**^	0.494
Rearfoot	94.2 (13.6)	58.4 (9.2)**	66.8 (9.6)**	74.6 (11.3)**	63.0 (10.3)**	< 0.001**	0.934

^**#**^Indicates *P* < 0.05, **indicate *P* < 0.001, significant difference from barefoot.

MPP indicates mean of peak pressure. PTI indicates pressure–time integral.

Significant values are in bold.

## Results

### Effect of insoles on dominant foot

There was no significant difference between dominant foot and subdominant foot (see Supplementary Table S2) on either MPP (*t*_216.359_ = 1.087, *P* = 0.343) or PTI (*t*_216.234_ = 0.328, *P* = 0.442) for diabetic elderly during gait. According to the one-way repeated measures ANOVA, the factor of the experimental design for the insoles had a significant effect on both the MPP (*F*(2.507,52.652) = 55.057, *P* < 0.001, ${\upeta }_{p}^{2}$= 0.724) and PTI (*F*(2.797,58.727) = 60.364, *P* = 0.004, ${\upeta }_{p}^{2}$= 0.742) on the dominant foot of the elderly subjects with Type 1 or 2 diabetes during gait. As the main ANOVA was significant, the pairwise comparison results (see Fig. 3) showed that both MPP (194.0 (38.6) kPa) and PTI (98.3 (12.9) kPa*s) with PORON Medical 4708 were the lowest, and significantly lower (*P* < 0.001) than with the other three conditions (i.e., barefoot, and use of Pe-Lite, and Nora Lunalight A fresh). The MPP (206.2 (30.2) kPa) and PTI (103.6 (25.7) kPa*s) values with the use of Nora Lunalastik EVA were the second lowest among the five conditions. The MPP (311.8 (66.0) kPa) and PTI (160.1 (27.9) kPa*s) values in the barefoot condition were the highest. Even though there was some reduction of pressure in comparison to the barefoot condition, the performance of Nora Lunalight A fresh and Pe-Lite was the most disappointing among the four insoles due to the higher MPP and PTI values.

### Effect of insoles on MPP in four different plantar regions

Table 2 shows that there is a significant effect from the insole on the forefoot (*F*(2.097, 44.033) = 93.702, *P* < 0.001, ${\upeta }_{p}^{2}$ = 0.934) and rearfoot (*F*(1.614,33.891) = 125.737, *P* < 0.001, ${\upeta }_{p}^{2}$ = 0.923). The MPP values in the barefoot condition were much more significantly higher for the forefoot (*P* < 0.001) and rearfoot (*P* < 0.001) than the other four insole conditions. On the other hand, the MPP of the toes in the barefoot condition was significantly lower (*F*(2.207,46.344) = 20.983, *P* < 0.001, ${\upeta }_{p}^{2}$ = 0.869) than when the insoles were worn.

Figure 4 shows the pairwise comparisons of the MPP in the four different plantar regions obtained by using the four different insoles while walking. Regardless of the plantar region, the MPP values of PORON Medical 4708 were all under 200 kPa. Among the four experimental insoles, the MPP values of PORON Medical 4708 were consistently lower than those of Nora Lunalight A fresh (toes, *P* < 0.001; forefoot, *P* < 0.001; midfoot, *P* = 0.003; and rearfoot, *P* < 0.001), Nora Lunalastik EVA (toes, *P* = 0.079; forefoot, *P* = 0.002; midfoot, *P* = 0.096; and rearfoot, *P* = 0.001), and Pe-Lite (toes, *P* < 0.001; forefoot, *P* < 0.001; midfoot, *P* < 0.001; and rearfoot, *P* < 0.001). The MPP of Nora Lunalastik EVA was the second lowest, and lower than that of Nora Lunalight A fresh (toes, *P* < 0.001; forefoot, *P* = 0.001; midfoot, *P* = 0.210; and rearfoot, *P* < 0.001) and Pe-Lite (toes, *P* < 0.001; forefoot, *P* = 0.007; midfoot, *P* = 0.234; and rearfoot, *P* < 0.001). The Nora Lunalight A fresh had the highest MPP values in the toes, forefoot, and rearfoot.

### Effect of insoles on PTI in four different plantar regions

It was found that experimental insoles had a significant impact on the PTI especially for the forefoot (*F*(1.857,39.001) = 61.558, *P* < 0.001, ${\upeta }_{p}^{2}$ = 0.845) and rearfoot *F*(2.116,44.444) = 85.857, *P* < 0.001, ${\upeta }_{p}^{2}$ = 0.934) when compared to the barefoot condition (Table 2). However, the PTI for the toes in the barefoot condition was significantly lower (*F*(2.395,50.298) = 33.635, *P* < 0.001, ${\upeta }_{p}^{2}$= 0.786) than when the four experimental insoles were worn.

Figure 5 shows the pairwise comparisons of the PTI in the four different plantar regions with the use of the four experimental insoles. Regardless of the plantar region, the PTI values of PORON Medical 4708 were the lowest among the four experimental insoles, and even lower than that of Nora Lunalight A fresh (toes, *P* < 0.001; forefoot, *P* < 0.001; midfoot, *P* = 0.022; and rearfoot, *P* < 0.001), Nora Lunalastik EVA (toes, *P* = 0.013; forefoot, *P* = 0.010; midfoot, *P* = 0.196; and rearfoot, *P* = 0.003), and Pe-Lite (toes, *P* < 0.001; forefoot, *P* < 0.001; midfoot, *P* = 0.003; and rearfoot, *P* < 0.001). The PTI value with the use of Nora Lunalastik EVA was the second lowest, but lower than that of Nora Lunalight A fresh (toes, *P* < 0.001; forefoot, *P* = 0.001; midfoot, *P* = 0.235; and rearfoot, *P* < 0.001) and Pe-Lite (toes, *P* = 0.017; forefoot, *P* = 0.003; midfoot, *P* = 0.160; and rearfoot, *P* = 0.098) in the four specific plantar regions.

### Subjective evaluation of four different insoles

In view of the perceived wear comfort of the insoles, PORON Medical 4708 received the highest score (7.3 (1.8)), Nora Lunalastik EVA the second highest (7.2 (1.7)), followed by Nora Lunalight A fresh and Pe-Lite (6.9 (1.7) and 6.8 (1.8), respectively). However, there was no significant difference among scores of the four insoles (*F*(2.090, 43.880) = 1.591, *P* = 0.060, ${\upeta }_{p}^{2}$ = 0.316).

## Discussion

The performance of contoured insoles made of different materials was systematically compared during practical use, thus providing a reference source on the type of insole materials to use for elderly with Type 1 or 2 diabetes in this study. The contoured insoles constructed of insole materials used in this study had significant effect on plantar pressure offloading. The MPP and PTI values were significantly reduced in the forefoot, and rearfoot regions by used insole materials as compared to the barefoot condition. The findings were consistent with hypotheses. The MPP and PTI values were significantly lower when contoured insoles fabricated from PORON Medical 4708, Pe-Lite, and Nora Lunalastik EVA were used in comparison to the barefoot condition. The main finding of this study was that the four experimental contoured insoles had significant effects on the offloading of the MPP and PTI in the forefoot and rearfoot when compared to the barefoot condition. The reduction of PTI in the heel, toes, and the forefoot are expected in diabetic patients wearing the appropriate diabetic footwear^21^. The literature has also shown that the peak pressure and PTIs in the forefoot and rearfoot are higher in diabetic patients especially with neuropathy^51^. Our study showed that the MPP and PTI values in the forefoot were the highest among the four different regions in the barefoot condition. Comparatively speaking, the MPP value of the forefoot was significantly reduced by 156.6 kPa (51.14%) to 99.1 kPa (32.4%), and the PTI was reduced from 51.1 kPa*s (44.8%) to 31.4 kPa*s (27.5%) when the four experimental contoured insoles were used. Meanwhile, the MPP values of the rearfoot were also reduced by 120.2 kPa (45.1%) to 74.2 kPa (27.8%), and PTI reduced by 35.8 kPa*s (38.0%) to 19.6 kPa*s (20.8%) with the use of four contoured insoles when compared to the barefoot condition. The orthotic insoles made of these soft cushioning foam materials act as shock absorbers to reduce shock transmission to the feet and joints. These findings indicate that the experimental insoles used in our study effectively off-load plantar pressure especially in the forefoot and rearfoot areas, thus the risk of DFUs can be reduced.

Contoured insoles offer adequate offloading for patients with diabetes. PORON is frequently recommended as an orthotic material for diabetic patients due to its superior ability to reduce plantar pressure^9^. The MPP value with the use of the PORON Medical 4708 insole was reduced by around 38.8% in the dominant foot, around 51.1% in the forefoot, and around 45.1% in the rearfoot when compared with the barefoot condition in this study. Birke et al.^11^ conducted a study on flat insoles showed that the PORON insole reduced the peak pressure by about 36–39%, while an insole composite constructed of a PORON Medical 4708 and a rigid foam material (i.e., Plastazote) reduced the MPP value in the forefoot by around 39%^31,32^. The reduction of MPP by PORON Medical 4708 in our study was larger than in previous studies, this could be attributed to the structure of the contoured insole with an embedded arch support. The finding is in line with previous studies^11,21,33,34^ that suggested an arch support may optimize the effect of plantar pressure offloading by insoles for diabetic feet. Therefore, due to the effectiveness in plantar pressure offloading by arch support, it is recommended to be incorporated as insole feature^52^.

In addition, the use of the PORON Medical 4708 insole resulted in a significantly lower MPP and PTI value in both the dominant foot and the four specific plantar regions (i.e., toes, forefoot, midfoot, and rearfoot) as opposed to the other three experimental insoles. And the MPP values of Nora Lunalastik EVA were the second lowest among four experimental insoles. The MPP values of PORON Medical 4708 were under 200 kPa—dominant foot (194.0 (38.6) kPa), and four specific plantar regions—180.6 (40.7) kPa (toes), 149.6 (28.6) kPa (forefoot), 84.0 (11.1) kPa (midfoot), and 146.4 (22.7) kPa (rearfoot), respectively. The MPP values of Nora Lunalastik EVA were under 200 kPa in four specific plantar regions other than dominant foot (206.2 (30.2) kPa). Waaijman et al.^53^ conducted a risk factor analysis of pressure-related plantar ulcer recurrence, found that the minor lesion index was only 0.4 for the risk of DFU recurrence when the peak pressure is under 200 kPa. This is in line with Bus^54^ study. Even though each diabetic patient has a different pressure threshold for DFU, Bus^54^ found that a planter pressure threshold value less than 200 kPa can be effective for plantar pressure offloading and DFU prevention. Owings et al.^55^ also suggested a pressure threshold of 207 kPa in the forefoot for footwear prescriptions for diabetic patients with a history of DFUs. The pressure redistribution of the PORON Medical 4708 insole in this study is in line with those of previous studies. Hence, the usage of soft insole materials especially PORON Medical 4708 has a high potential to reduce the DFU risk since the pressure values were below 200 kPa in this study.

The findings of this study showed that insole material properties significantly influenced the plantar pressure offloading performance of contoured insoles. The offloading performance of softness of insole material (i.e., PORON Medical 4708, and Nora Lunalastik EVA) had better performance of plantar pressure offloading than rigid insole materials (i.e., Nora Lunalight A fresh, and Pe-Lite) in four plantar regions. This might be due to their extra softness (hardness of 18 Shore A) which facilitates force absorption during gait^31^. The usage of Polyurethane (i.e., PORON Medical 4708) in orthopaedic insoles not only provides resistance to ground reaction forces during gait, but also high resistance to bottoming out under pressure, shock and shear due to its good cushioning and pressure redistribution properties^30^. Soft material with lower density of Nora Lunalastik EVA (hardness of 23 Shore A) performed better than the rigid material with higher density of Nora Lunalight A fresh (hardness of 58 Shore A) on offloading plantar pressure in this study. The result is in line with the finding of Tang et al.^56^, low density ethylene–vinyl acetate (i.e., Nora Lunalastik EVA) is generally soft and provides good cushioning, shock absorption during walk^30^. In general, the softness of insole material relates with better performance on plantar pressure offloading in this study. Although there was no significant difference in scoring among the four experimental insoles, the evidence shows in this study that soft, highly compressive insole material has the best offloading performance. The findings in this study can be referenced by medical footcare industries and address the research gap of contoured insole studies that involve the elderly with Type 1 or 2 diabetes. The MPP and PTI values were higher in toes region with four insoles when compared to barefoot condition due to the extra pressure induced by shoes wearing in study. The limitations of this study are the effectiveness of pressure offloading on long-term wear by utilizing experimental contoured insoles shall be further studied, and sample size is limited. In addition, the performance of used insoles on plantar pressure offloading in diabetic elderly with Type 1 or 2 who have a diagnosis of distal neuropathy or foot deformity is also needed for further study.

## Conclusions

The offloading performance of contoured insoles made of different insole materials has been systematically examined in this study, thus providing scientific evidence and guidance on the usage of insole materials for the elderly who suffer from Type 1 and 2 diabetes in this study. The contoured insoles used in this study significantly offloaded the MPP and PTI values in the forefoot, and rearfoot regions of the dominant foot as compared to the barefoot condition. The soft insole materials (i.e., PORON medical 4708 and Nora Lunalastik EVA) had a better performance than the rigid insole materials (i.e., Nora Lunalight A fresh, and Pe-Lite) on plantar pressure offloading. The PORON Medical 4708 contoured insole had the best performance for plantar pressure offloading across the four different plantar regions. The findings of this study could provide practical information for clinicians to better understand various insole materials, thus prescribe suitable insoles for the elderly with diabetes.

## Supplementary Information

Supplementary Table S1.

Supplementary Table S2.

## Data Availability

Data are available upon reasonable request. If you want to request the data from this study, please contact corresponding author, Dr. Kit-lun Yick.

## References

[1] Federation, I. D. *Diabetes around the world in 2021*. https://diabetesatlas.org/ (2021).

[2] Asiimwe D, Mauti GO, Kiconco R (2020). Prevalence and risk factors associated with type 2 diabetes in elderly patients aged 45–80 years at Kanungu District. J. Diabetes Res..

[3] Duan Y (2021). The effects of different accumulated pressure-time integral stimuli on plantar blood flow in people with diabetes mellitus. BMC Musculoskelet. Disord..

[4] Armstrong DG, Boulton AJ, Bus SA (2017). Diabetic foot ulcers and their recurrence. N. Engl. J. Med..

[5] Yap, M. H. *et al.* in *2021 IEEE EMBS International Conference on Biomedical and Health Informatics (BHI).* 1–4 (IEEE).

[6] Pereiro-Buceta H (2021). The effect of simulated leg-length discrepancy on the dynamic parameters of the feet during gait: Cross-sectional research. Healthcare.

[7] Bus S, Waaijman R (2013). The value of reporting pressure–time integral data in addition to peak pressure data in studies on the diabetic foot: A systematic review. Clin. Biomech..

[8] Lu Y, Mei Q, Gu Y (2015). Plantar loading reflects ulceration risks of diabetic foot with toe deformation. BioMed Res. Int..

[9] Gerrard JM, Bonanno DR, Whittaker GA, Landorf KB (2020). Effect of different orthotic materials on plantar pressures: A systematic review. J. Foot Ankle Res..

[10] Abri H (2019). Plantar pressure distribution in diverse stages of diabetic neuropathy. J. Diabetes Metab. Disord..

[11] Birke JA, Foto JG, Pfiefer LA (1999). Effect of orthosis material hardness on walking pressure in high-risk diabetes patients. JPO.

[12] Fernando, M., Wearing, S. & Crowther, R. The importance of foot pressure in diabetes. In *Handbook of Human Motion* 1–29 (2016).

[13] Zhang B, Lu Q (2020). A current review of foot disorder and plantar pressure alternation in the elderly. Phys. Act. Health.

[14] Barn R, Waaijman R, Nollet F, Woodburn J, Bus SA (2015). Predictors of barefoot plantar pressure during walking in patients with diabetes, peripheral neuropathy and a history of ulceration. PLoS ONE.

[15] McCartan BL, Rosenblum BI (2014). Offloading of the diabetic foot: Orthotic and pedorthic strategies. Clin. Podiatr. Med. Surg..

[16] Nouman M, Dissaneewate T, Leelasamran W, Chatpun S (2019). The insole materials influence the plantar pressure distributions in diabetic foot with neuropathy during different walking activities. Gait Posture.

[17] Chicharro-Luna E (2020). Predictive model to identify the risk of losing protective sensibility of the foot in patients with diabetes mellitus. Int. Wound J..

[18] Navarro-Flores E (2020). The reliability, validity, and sensitivity of the Edmonton Frail Scale (EFS) in older adults with foot disorders. Aging (Albany NY).

[19] Bôas NCRV, Salomé GM, Ferreira LM (2018). Frailty syndrome and functional disability among older adults with and without diabetes and foot ulcers. J. Wound Care.

[20] Fernando ME (2015). Lower limb biomechanical characteristics of patients with neuropathic diabetic foot ulcers: The diabetes foot ulcer study protocol. BMC Endocr. Disord..

[21] Hsi W-L, Chai H-M, Lai J-S (2002). Comparison of pressure and time parameters in evaluating diabetic footwear. Am. J. Phys. Med. Rehabil..

[22] Westra M, van Netten JJ, Manning HA, van Baal JG, Bus SA (2018). Effect of different casting design characteristics on offloading the diabetic foot. Gait Posture.

[23] Chapman J (2013). Effect of rocker shoe design features on forefoot plantar pressures in people with and without diabetes. Clin. Biomech..

[24] Keukenkamp R, Busch-Westbroek T, Barn R, Woodburn J, Bus S (2021). Foot ulcer recurrence, plantar pressure and footwear adherence in people with diabetes and Charcot midfoot deformity: A cohort analysis. Diabet. Med..

[25] Bonanno DR (2019). Effects of a contoured foot orthosis and flat insole on plantar pressure and tibial acceleration while walking in defence boots. Sci. Rep..

[26] Martinez-Santos A, Preece S, Nester CJ (2019). Evaluation of orthotic insoles for people with diabetes who are at-risk of first ulceration. J. Foot Ankle Res..

[27] Ahmed S, Barwick A, Butterworth P, Nancarrow S (2020). Footwear and insole design features that reduce neuropathic plantar forefoot ulcer risk in people with diabetes: A systematic literature review. J. Foot Ankle Res..

[28] Bus SA (2016). Footwear and offloading interventions to prevent and heal foot ulcers and reduce plantar pressure in patients with diabetes: A systematic review. Diabetes Metab. Res. Rev..

[29] Janisse DJ (2005). The therapeutic shoe bill: Medicare coverage for prescription footwear for diabetic patients. Foot Ankle Int..

[30] Yick, K.-L. & Tse, C.-Y. in *Handbook of Footwear Design and Manufacture* 361–388 (Elsevier, 2021).

[31] Tong JW, Ng EY (2010). Preliminary investigation on the reduction of plantar loading pressure with different insole materials (SRP–Slow Recovery Poron®, P-Poron®, PPF–Poron®+ Plastazote, firm and PPS–Poron®+ Plastazote, soft). Foot.

[32] Rogers K, Otter S, Birch I (2006). The effect of PORON® and Plastazote® insoles on forefoot plantar pressures. Br. J. Podiatry.

[33] Arts M (2015). Data-driven directions for effective footwear provision for the high-risk diabetic foot. Diabet. Med..

[34] Guldemond N (2007). The effects of insole configurations on forefoot plantar pressure and walking convenience in diabetic patients with neuropathic feet. Clin. Biomech..

[35] Owings TM, Woerner JL, Frampton JD, Cavanagh PR, Botek G (2008). Custom therapeutic insoles based on both foot shape and plantar pressure measurement provide enhanced pressure relief. Diabetes Care.

[36] San Tsung BY, Zhang M, Mak AFT, Wong MWN (2004). Effectiveness of insoles on plantar pressure redistribution. J. Rehabil. Res. Dev..

[37] Stief T, Peikenkamp K (2015). A new insole measurement system to detect bending and torsional moments at the human foot during footwear condition: A technical report. J. Foot Ankle Res..

[38] Lo WT (2014). New methods for evaluating physical and thermal comfort properties of orthotic materials used in insoles for patients with diabetes. J. Rehabil. Res. Dev..

[39] Kermen E (2020). New Approach to Foot Orthotics Materials: Hydrogel Insoles.

[40] Alsubiheen A, Petrofsky J, Daher N, Lohman E, Balbas E (2015). Effect of tai chi exercise combined with mental imagery theory in improving balance in a diabetic and elderly population. Med. Sci. Monit. Int. Med. J. Exp. Clin. Res..

[41] Sacco IC (2014). Abnormalities of plantar pressure distribution in early, intermediate, and late stages of diabetic neuropathy. Gait Posture.

[42] Raspovic A (2013). Gait characteristics of people with diabetes-related peripheral neuropathy, with and without a history of ulceration. Gait Posture.

[43] Sawacha Z (2012). Integrated kinematics–kinetics–plantar pressure data analysis: A useful tool for characterizing diabetic foot biomechanics. Gait Posture.

[44] Carbone F, Portincasa P, Frühbeck G, Nathoe HM (2021). Endocrinology and Systemic Diseases.

[45] Kwan M-Y, Yick K-L, Yip J, Tse C-Y (2021). The immediate effects of hallux valgus orthoses: A comparison of orthosis designs. Gait Posture.

[46] Lin TL (2013). The effect of removing plugs and adding arch support to foam based insoles on plantar pressures in people with diabetic peripheral neuropathy. J. Foot Ankle Res..

[47] Raspovic A, Landorf KB, Gazarek J, Stark M (2012). Reduction of peak plantar pressure in people with diabetes-related peripheral neuropathy: An evaluation of the DH Pressure Relief Shoe. J. Foot Ankle Res..

[48] Duan Y (2022). Relationship between plantar tissue hardness and plantar pressure distributions in people with diabetic peripheral neuropathy. Front. Bioeng. Biotechnol..

[49] Cham MB, Mohseni-Bandpei MA, Bahramizadeh M, Kalbasi S, Biglarian A (2018). The effects of vibro-medical insole on sensation and plantar pressure distribution in diabetic patients with mild-to-moderate peripheral neuropathy. Clin. Biomech. (Bristol, Avon).

[50] Seeley MK, Umberger BR, Shapiro R (2008). A test of the functional asymmetry hypothesis in walking. Gait Posture.

[51] Fernando M (2013). Biomechanical characteristics of peripheral diabetic neuropathy: A systematic review and meta-analysis of findings from the gait cycle, muscle activity and dynamic barefoot plantar pressure. Clin. Biomech..

[52] Collings R, Freeman J, Latour JM, Paton J (2021). Footwear and insole design features for offloading the diabetic at risk foot: A systematic review and meta-analyses. Endocrinol. Diabetes Metab..

[53] Waaijman R (2014). Risk factors for plantar foot ulcer recurrence in neuropathic diabetic patients. Diabetes Care.

[54] Bus SA (2016). The role of pressure offloading on diabetic foot ulcer healing and prevention of recurrence. Plast. Reconstr. Surg..

[55] Owings T (2009). Plantar pressures in diabetic patients with foot ulcers which have remained healed. Diabet. Med..

[56] Tang UH (2014). Comparison of plantar pressure in three types of insole given to patients with diabetes at risk of developing foot ulcers: A two-year, randomized trial. J. Clin. Transl. Endocrinol..

