# Supplementary material for: Effects of contoured insoles with different materials on plantar pressure offloading in diabetic elderly during gait
Source: Sci Rep. 2022 Sep 13;12:15395. doi: 10.1038/s41598-022-19814-0 (PMC9470545; doi:10.1038/s41598-022-19814-0)
Supplement: Supplementary file 1 — Supplementary Table S1. [file 41598_2022_19814_MOESM1_ESM.docx]

Table 1 Randomized order of experimental protocol

| Items | Order 1 | Order 2 | Order 3 | Order 4 | Order 5 |
| --- | --- | --- | --- | --- | --- |
| Participant 1 | PORON Medical 4708 | Nora Lunalight A fresh | Pe-Lite | Barefoot | Nora Lunalastik EVA |
| Participant 2 | Barefoot | Nora Lunalastik EVA | PORON Medical 4708 | Pe-Lite | Nora Lunalight A fresh |
| Participant 3 | Barefoot | Nora Lunalight A fresh | PORON Medical 4708 | Pe-Lite | Nora Lunalastik EVA |
| Participant 4 | Nora Lunalight A fresh | Barefoot | Nora Lunalastik EVA | Pe-Lite | PORON Medical 4708 |
| Participant 5 | Pe-Lite | Nora Lunalastik EVA | PORON Medical 4708 | Barefoot | Nora Lunalight A fresh |
| Participant 6 | PORON Medical 4708 | Pe-Lite | Nora Lunalastik EVA | Nora Lunalight A fresh | Barefoot |
| Participant 7 | Pe-Lite | Barefoot | Nora Lunalastik EVA | Nora Lunalight A fresh | PORON Medical 4708 |
| Participant 8 | Pe-Lite | PORON Medical 4708 | Nora Lunalastik EVA | Nora Lunalight A fresh | Barefoot |
| Participant 9 | Nora Lunalastik EVA | Pe-Lite | PORON Medical 4708 | Barefoot | Nora Lunalight A fresh |
| Participant 10 | Nora Lunalight A fresh | PORON Medical 4708 | Barefoot | Pe-Lite | Nora Lunalastik EVA |
| Participant 11 | Barefoot | Pe-Lite | Nora Lunalastik EVA | PORON Medical 4708 | Nora Lunalight A fresh |
| Participant 12 | Nora Lunalastik EVA | Nora Lunalight A fresh | Pe-Lite | Barefoot | PORON Medical 4708 |
| Participant 13 | Barefoot | Nora Lunalight A fresh | Pe-Lite | PORON Medical 4708 | Nora Lunalastik EVA |
| Participant 14 | Pe-Lite | PORON Medical 4708 | Nora Lunalastik EVA | Nora Lunalight A fresh | Barefoot |
| Participant 15 | Nora Lunalight A fresh | Pe-Lite | Nora Lunalastik EVA | Barefoot | PORON Medical 4708 |
| Participant 16 | Nora Lunalight A fresh | Barefoot | PORON Medical 4708 | Pe-Lite | Nora Lunalastik EVA |
| Participant 17 | Barefoot | Nora Lunalastik EVA | PORON Medical 4708 | Pe-Lite | Nora Lunalight A fresh |
| Participant 18 | Barefoot | Pe-Lite | Nora Lunalastik EVA | PORON Medical 4708 | Nora Lunalight A fresh |
| Participant 19 | Nora Lunalastik EVA | Pe-Lite | PORON Medical 4708 | Nora Lunalight A fresh | Barefoot |
| Participant 20 | Barefoot | PORON Medical 4708 | Pe-Lite | Nora Lunalight A fresh | Nora Lunalastik EVA |
| Participant 21 | Pe-Lite | Nora Lunalastik EVA | PORON Medical 4708 | Nora Lunalight A fresh | Barefoot |
| Participant 22 | Barefoot | Nora Lunalastik EVA | Pe-Lite | PORON Medical 4708 | Nora Lunalight A fresh |
