# Supplementary material for: Effects of contoured insoles with different materials on plantar pressure offloading in diabetic elderly during gait
Source: Sci Rep. 2022 Sep 13;12:15395. doi: 10.1038/s41598-022-19814-0 (PMC9470545; doi:10.1038/s41598-022-19814-0)
Supplement: Supplementary file 2 — Supplementary Table S2. [file 41598_2022_19814_MOESM2_ESM.docx]

Table 2. Statistical results of MPP and PTI with and without insoles of subdominant foot (mean (SD))

| **Plantar region** | | Barefoot | PORON Medical 4708 | Pe-Lite | Nora Lunalight A fresh | Nora Lunalastik EVA | P value | $\eta_{p}^{2}$ |
| --- | --- | --- | --- | --- | --- | --- | --- | --- |
| **MPP**  (kPa) | Toes | 163.3 (54.4) | 169.1 (37.4) | 207.0 (38.8) **^#^** | 236.5 (62.0)** | 184.2 (37.7) | **<0.001**** | 0.434 |
|  | Forefoot | 299.0 (48.4) | 153.2 (27.5)** | 189.7 (40.1)** | 204.7 (58.8)** | 168.4 (24.6)** | **<0.001**** | 0.851 |
|  | Midfoot | 102.4 (54.6) | 82.7 (12.4) | 94.6 (22.4) | 95.5 (23.8) | 88.5 (17.1) | 0.085 | 0.125 |
|  | Rearfoot | 256.9 (60.8) | 146.9 (16.7)** | 172.4 (18.8)** | 192.3 (21.0)** | 163.6 (19.1)** | **<0.001**** | 0.732 |
| **PTI**  (kPa*s) | Toes | 52.1 (18.7) | 64.2 (14.7) **^#^** | 74.6 (16.9) ** | 86.7 (28.0) ** | 70.6 (15.2) ** | **<0.001**** | 0.531 |
|  | Forefoot | 111.3 (24.2) | 64.6 (11.5) ** | 76.1 (17.7) ** | 81.5 (18.3) ** | 70.2 (11.3) ** | **<0.001**** | 0.797 |
|  | Midfoot | 44.2 (22.7) | 45.9 (10.6) | 50.2 (14.4) | 52.5 (15.6) | 47.6 (10.8) | **0.039^#^** | 0.157 |
|  | Rearfoot | 94.5 (20.3) | 60.5 (12.4)** | 69.3 (14.2) **^#^** | 79.0 (14.1) **^#^** | 72.3 (28.2) **^#^** | **<0.001**** | 0.432 |

Notes: **^#^** indicates *P* < 0.05, ** indicate *P* < 0.001, significant difference from barefoot.

MPP indicates mean of peak pressure. PPT indicates pressure-time integral.
